# Chinese Oncologists’ Perspectives on Integrating AI into Clinical Practice: Cross-Sectional Survey Study

**DOI:** 10.2196/53918

**Published:** 2024-06-05

**Authors:** Ming Li, XiaoMin Xiong, Bo Xu, Conan Dickson

**Affiliations:** 1 Department of Health Policy Management Bloomberg School of Public Health Johns Hopkins University Baltimore, MD United States; 2 Chongqing Key Laboratory of Intelligent Oncology for Breast Cancer Chongqing University Cancer Hospital Chongqing University School of Medicine Chongqing China; 3 Department of Biochemistry and Molecular Biology, Key Laboratory of Breast Cancer Prevention and Therapy Ministry of Education, National Cancer Research Center Tianjin Medical University Cancer Institute and Hospital Tianjin China

**Keywords:** artificial intelligence, AI, machine learning, oncologist, concern, clinical practice

## Abstract

**Background:**

The rapid development of artificial intelligence (AI) has brought significant interest to its potential applications in oncology. Although AI-powered tools are already being implemented in some Chinese hospitals, their integration into clinical practice raises several concerns for Chinese oncologists.

**Objective:**

This study aims to explore the concerns of Chinese oncologists regarding the integration of AI into clinical practice and to identify the factors influencing these concerns.

**Methods:**

A total of 228 Chinese oncologists participated in a cross-sectional web-based survey from April to June in 2023 in mainland China. The survey gauged their worries about AI with multiple-choice questions. The survey evaluated their views on the statements of “The impact of AI on the doctor-patient relationship” and “AI will replace doctors.” The data were analyzed using descriptive statistics, and variate analyses were used to find correlations between the oncologists’ backgrounds and their concerns.

**Results:**

The study revealed that the most prominent concerns were the potential for AI to mislead diagnosis and treatment (163/228, 71.5%); an overreliance on AI (162/228, 71%); data and algorithm bias (123/228, 54%); issues with data security and patient privacy (123/228, 54%); and a lag in the adaptation of laws, regulations, and policies in keeping up with AI’s development (115/228, 50.4%). Oncologists with a bachelor’s degree expressed heightened concerns related to data and algorithm bias (34/49, 69%; *P*=.03) and the lagging nature of legal, regulatory, and policy issues (32/49, 65%; *P*=.046). Regarding AI’s impact on doctor-patient relationships, 53.1% (121/228) saw a positive impact, whereas 35.5% (81/228) found it difficult to judge, 9.2% (21/228) feared increased disputes, and 2.2% (5/228) believed that there is no impact. Although sex differences were not significant (*P*=.08), perceptions varied—male oncologists tended to be more positive than female oncologists (74/135, 54.8% vs 47/93, 50%). Oncologists with a bachelor’s degree (26/49, 53%; *P*=.03) and experienced clinicians (≥21 years; 28/56, 50%; *P*=.054). found it the hardest to judge. Those with IT experience were significantly more positive (25/35, 71%) than those without (96/193, 49.7%; *P*=.02). Opinions regarding the possibility of AI replacing doctors were diverse, with 23.2% (53/228) strongly disagreeing, 14% (32/228) disagreeing, 29.8% (68/228) being neutral, 16.2% (37/228) agreeing, and 16.7% (38/228) strongly agreeing. There were no significant correlations with demographic and professional factors (all *P*>.05).

**Conclusions:**

Addressing oncologists’ concerns about AI requires collaborative efforts from policy makers, developers, health care professionals, and legal experts. Emphasizing transparency, human-centered design, bias mitigation, and education about AI’s potential and limitations is crucial. Through close collaboration and a multidisciplinary strategy, AI can be effectively integrated into oncology, balancing benefits with ethical considerations and enhancing patient care.

## Introduction

Artificial intelligence (AI) has made substantial strides within the health care sector, effecting profound transformations in various fields including medicine, radiology, dermatology, ophthalmology, and pathology. The potential of AI to reform physicians’ clinical practices is significant [1].

AI unveils a plethora of opportunities within health care, demonstrating capabilities to augment a host of medical processes—from disease diagnostics and chronic disease management to clinical decision-making. With AI becoming increasingly ubiquitous, its utility in enhancing the accuracy and efficiency of clinical practice across a multitude of specializations is clear [2]. Particularly in the field of oncology, AI is revolutionizing practice paradigms, offering crucial advancements in the management of patients with cancer [3].

The proliferation of data and advances in computational algorithms have positioned AI to ameliorate clinical oncology via rigorously evaluated, narrow-task applications interacting at specific touch points along the cancer care path [4]. This, in turn, has expedited progress in oncology research, enhancing cancer diagnosis and treatment.

The concept of intelligent oncology was introduced as an emerging field that integrates various disciplines including oncology, radiology, pathology, molecular biology, multiomics, and computer science. This integration aims to leverage data and computational methods to improve cancer care and outcomes [5].

In China, approximately 32.86% of hospitals have adopted 1 or more AI products, with all university hospitals having integrated AI technologies [6]. These technologies are primarily used in imaging AI and clinical decision support systems across breast cancer, bone tumors, urological tumors, and many other types of cancer [7-12].

The AI Decision System was established under the Chinese Society of Clinical Oncology platform using databases, guidelines, and technologies. The main goal of the system is to provide patients with breast cancer with more accurate and individualized medical decisions, and the system has been validated effectively in clinical trials and implemented in many hospitals in China [13].

Differences in viewpoints across various specialties and demographic groups could significantly influence the speed and effectiveness of AI adoption. Distinct perspectives based on sex and age have been observed regarding AI [14]. It is crucial to have a comprehensive understanding of these differences to ensure the priorities of all stakeholders are taken into account. In China, especially among oncologists, there is a lack of research exploring physicians’ attitudes toward AI. Given that physicians are the main users of AI technologies, their perspectives and concerns need to be meticulously addressed.

Although most physicians recognize the potential benefits of AI in health care, some maintain a cautious stance toward its adoption [15]. Interestingly, about 41% of physicians find themselves equally excited and concerned about the possibilities that AI presents in the health care sector [16]. The effect of AI on patient outcomes is still uncertain. Significant obstacles to the adoption of AI in this field include issues with biased and heterogeneous data, challenges in data management, and others [1].

The primary aim of this study is to delineate oncologists’ concerns surrounding AI. Understanding these concerns can inform strategies to foster AI acceptance and adoption in clinical practice, thereby optimizing patient outcomes. The insights derived from this study can provide valuable guidance to policy makers and regulatory bodies, assisting in comprehending AI’s current use, gauging its impact, identifying potential risks, and determining requisite regulations to ensure ethical and effective AI use. Moreover, these insights can aid AI firms in fine-tuning their products to better align with physicians’ needs, thereby increasing the practicality and utility of AI tools in clinical practice [17].

## Methods

### Study Design

The development of this questionnaire (Multimedia Appendix 1) was grounded in an extensive literature review, complemented by interviews with 11 oncologists. Before the survey’s deployment, these oncologists, who are specialists in various domains of cancer treatment such as medical, surgical, and radiation oncology and have experience with AI technologies in contexts such as medical imaging analysis and treatment recommendations, provided valuable feedback.

The inclusion criteria for participation in the study were limited to licensed oncologists currently practicing in Chinese hospitals and actively treating patients with cancer. The exclusion criteria ruled out general practitioners, general surgeons, medical residents, students, and other health care professionals such as nurses or technicians. Only attending physicians specializing in oncology who are seeing patients in Chinese hospitals were eligible to participate.

Using WeChat (Tencent), a popular communication tool in China, for survey distribution ensured a streamlined and effective process for collecting data. The survey was conducted from April 4 to June 30, 2023, and was distributed across the country by the Chinese Anti-Cancer Association.

The questionnaire, presented in Chinese, was structured around 4 main components. The first section concentrated on the oncologists’ characteristics. The second section encompassed questions pertaining to their knowledge and perception of AI. The third section probed the promoting factors for the use of AI. The final section aimed to explore their concerns regarding AI. All question items were mandatory; otherwise, a response cannot be submitted successfully, which ensured that no data were lost or missing.

The main objective of the study was to explore into oncologists’ concern on AI. The survey was anchored by 3 principal questions, 1 of which focused on concerns about AI, providing 10 options for multiple-choice answers along with a free-text option for detailed responses. The impact of AI on the doctor-patient relationship was assessed through 4 predefined options: positive, negative, no impact, and hard to judge. Furthermore, the survey measured participants’ views on the assertion that “AI will replace doctors” using a 5-point Likert scale.

### Ethical Considerations

The ChongQing University Cancer Hospital’s Institutional Review Board approved the study (CZLS2022244-A). An electronic consent form was presented on the initial page of the questionnaire. Only participants who agreed to this consent form could continue to answer the questionnaire. Participation in this survey was entirely voluntary and anonymous, and data were deidentified. No compensation was provided for participation.

### Data Analysis

Descriptive statistics were used to summarize the survey findings, notably the ranking of oncologists’ concerns. The association between physicians’ characteristics and their AI-related concerns was evaluated using the *χ*^2^ test. To scrutinize the variation in responses, factors such as the oncologists’ sex, education, and years of experience were examined using the Pearson *χ*^2^ test. The statistical significance of the analysis was ascertained using 2-sided testing with an α level of 5%.

A *P* value of less than .05 was considered to be indicative of statistical significance. All data analyses were performed using SPSS software (version 22.0; IBM Corp).

## Results

### Oncologists’ Characteristics

Our study involved a sample of 228 oncologists. The majority were male (n=135, 59.2%), whereas female participants constituted 40.8% (n=93). The largest age group was 31-40 years (n=95, 41.7%), followed by 41-50 years (n=80, 35.1%), younger than 30 years (n=28, 12.3%), and 51-60 years (n=25, 11%). Regarding years of clinical practice, the most represented group had 11-20 years of experience (n=126, 55.3%), compared to those with 0-10 years (n=49, 21.5%) and over 20 years (n=53, 23.2%). In terms of education, the largest proportion held a bachelor’s degree (n=89, 39%), with fewer having master’s (n=83, 36.4%) or doctoral degrees (n=56, 24.6%). Medical oncology was the most common specialty (n=97, 42.5%), followed by surgical oncology (n=77, 33.8%), radiation therapy (n=40, 17.5%), and other specialties (n=14, 6.1%) such as Chinese traditional medicine oncologists and gynecologic oncologists. Most oncologists worked in university hospitals (n=148, 64.9%), whereas others worked in nonuniversity hospitals (n=80, 35.1%). Experience with IT projects was limited, with only 15.4% (n=35) having such experience, compared to 84.6% (n=193) without (Table 1).

**Table 1:** Oncologists’ characteristics (N=228).

Characteristics		Oncologists, n (%)
**Sex**		
	Male	135 (59.2)
	Female	93 (40.8)
**Years of clinical practice**		
	0-10	49 (21.5)
	11-20	126 (55.3)
	≥21	53 (23.2)
**Education degree**		
	Bachelor’s	89 (39)
	Master’s	83 (36.4)
	Doctoral	56 (24.6)
**Specialty**		
	Medical oncology	97 (42.5)
	Surgical oncology	77 (33.8)
	Radiation therapy	40 (17.5)
	Others	14 (6.1)
**Hospital type**		
	University hospital	148 (64.1)
	Nonuniversity hospital	80 (35.1)
**Experience with IT projects**		
	Yes	35 (15.4)
	No	193 (84.6)

### Oncologists’ Concern About AI

Respondents expressed their level of concern regarding aspects of implementing AI in health care, with key findings summarized based on the analysis of recurrent themes in their selection of responses from the available multiple-choice options (Table 2).

From the 228 respondents, the most prominent concern related to the risk that AI could mislead physicians’ diagnosis and treatment, causing medical errors and impacting patient safety (n=163, 71.5%), followed closely by the potential decrease in physicians’ diagnostic and therapeutic capabilities due to an overreliance on AI (n=162, 71%).

Concerns about data bias and inapplicability of AI to actual clinical situations were expressed by 54% (n=123) of the respondents, tying with worries about data security and patient privacy. Legal and regulatory lagging were prominent issues for 50.4% (n=115) of the respondents.

The “black box” phenomenon and lack of trust were cited as problems by 39.5% (n=90) of the respondents. The lack of empathy in AI, demonstrating a deficiency in humanlike emotions, was a concern for 36.8% (n=84) of the physicians. Issues related to the pricing of AI products and their impact on widespread use were pointed out by 25.4% (n=58) of respondents, and 16.7% (n=38) found the operation of AI products complex and not well integrated within existing workflow.

Interestingly, only 3.1% (n=7) of the physicians felt no concern associated with the application of AI, and a very small percentage (n=2, 0.9%) marked “other” concerns.

Supplementary analyses were executed to discern potential variations in AI concerns, based on physicians’ demographic and professional traits. These analyses considered the sex, age, education level, years of clinical practice, area of specialty, hospital type, and IT experience of the participating clinicians (Tables 3 and 4).

As showed in first rows of Tables 3 and 4, regarding sex, our data revealed no statistically significant differences between male and female oncologists in their perceptions of AI in the health care context (all *P*>.05). Overall, 71.8% (95/135) of male physicians and 71% (66/93) of female physicians were concerned about AI misleading diagnosis and treatment, whereas the concerns about an overreliance on AI were shared by 69.6% (94/135) of male physicians and 73% (68/93) of female physicians.

When examining education level, oncologists holding a bachelor’s degree were more likely to be concerned about data and algorithm bias (34/49, 69%; *P*=.03) and laws, regulation, and policies lagging (32/49, 65%; *P*=.046).

Considering the years of clinical practice, oncologists with 0-10 years of experience exhibited less concern about laws, regulations, and policies lagging behind (37/89, 42%; *P*=.047).

Regarding the clinician’s area of specialty, no significant differences were detected in their concerns about AI (all *P*>.05).

In terms of hospital type, there was a trend toward a greater concern about the business model issue of AI services among clinicians working at university hospitals, although this did not reach statistical significance (17/58, 29%; *P*=.09).

Lastly, with regard to IT experience, clinicians with such experience were found to express significantly less concern on an overreliance on AI (19/35, 54%; *P*=.02) and lower levels of empathy (12/84, 14%; *P*=.003) in comparison to those without IT experience.

**Table 2:** Oncologists’ concerns about AI^a^ (N=228).

Concerns	Oncologists, n (%)
AI misleads diagnosis and treatment	163 (71.5)
Overreliance on AI	162 (71)
Data and algorithm bias	123 (54)
Data security and patient privacy issues	123 (54)
Laws, regulation, and policies lagging	115 (50.4)
“Black box” phenomenon	90 (39.5)
AI has no empathy and lacks human emotions	84 (36.8)
Business model issues affect AI promotion	58 (25.4)
Not easy to use and not well integrated with clinical workflow	38 (16.7)
No concern	7 (3.1)
Others	2 (0.9)

^a^AI: artificial intelligence.

**Table 3:** Oncologists’ characteristics in relation to their concerns about AI^a^ (part 1).

Characteristics		Total oncologists, N	AI misleads diagnosis and treatment						Overreliance on AI							Data and algorithm bias				
			Oncologists, n (%)^b^	Chi-square (*df*)		*P* value		Oncologists, n (%)		Chi-square (*df*)		*P* value		Oncologists, n (%)			Chi-square (*df*)		*P* value	
**Sex**					0.021 (1)		.88				0.326 (1)		.57					0.05 (1)		.82
	Male	135	97 (71.8)					94 (69.6)						72 (53.3)						
	Female	93	66 (71)					68 (73.1)						51 (54.8)						
**Education degree**					1.827 (2)		.40				1.665 (2)		.44					6.746 (2)		.03^c^
	Bachelor’s	49	36 (73.5)					38 (77.6)						34 (69.4)						
	Master’s	126	93 (73.8)					89 (70.6)						60 (47.6)						
	Doctoral	53	34 (64.2)					35 (66)						29 (54.7)						
**Years of clinical practice**					5.187 (2)		.08				3.556 (2)		.17					2.634 (2)		.27
	0-10	89	67 (75.3)					57 (64)						50 (56.2)						
	11-20	83	52 (62.6)					62 (74.7)						48 (57.8)						
	≥21	56	44 (78.6)					43 (76.8)						25 (44.6)						
**Specialty**					1.769 (3)		.62				1.242 (3)		.74					3.6 (3)		.31
	Medical oncology	97	67 (69.1)					66 (68)						56 (57.7)						
	Surgical oncology	77	54 (70.1)					55 (71.4)						35 (45.4)						
	Radiation therapy	40	32 (80)					31 (77.5)						23 (57.5)						
	Others	14	10 (71.4)					10 (71.4)						9 (64.3)						
**Hospital type**					0.563 (1)		.90				0.756 (1)		.38					1.34 (1)		.25
	University hospital	148	41 (76.5)					108 (73)						84 (56.8)						
	Nonuniversity hospital	80	56 (69.7)					54 (67.5)						39 (48.8)						
**IT experience**					0.158 (1)		.69				5.651 (1)		.02^c^					1.321 (1)		.25
	Yes	35	26 (74.3)					19 (54.3)						22 (62.9)						
	No	193	137 (71)					143 (74.1)						101 (52.3)						

^a^AI: artificial intelligence.

^b^Percentages expressed with the value in the “Total oncologists, N” column as the denominator.

^c^*P*<.05.

**Table 4:** Oncologists’ characteristics in relation to their concerns about AI^a^ (part 2).

Characteristics			Total oncologists, N		Data security and patient privacy issues						Laws, regulation, and policies lagging							“Black box” phenomenon				
				Oncologists, n (%)^b^		Chi-square (*df*)		*P* value		Oncologists, n (%)		Chi-square (*df*)		*P* value		Oncologists, n (%)			Chi-square (*df*)		*P* value	
**Sex**							0.1 (1)		.75				0.614 (1)		.43					0.326 (1)		.57
	Male	135		74 (54.8)						71 (52.6)						94 (69.6)						
	Female	93		49 (52.7)						44 (47.3)						68 (73.1)						
**Education degree**							0.634 (2)		.73				6.149 (2)		.046^c^					0.106 (2)		.95
	Bachelor’s	49		28 (57.1)						32 (65.3)						20 (40.8)						
	Master’s	126		65 (51.6)						56 (44.4)						50 (39.7)						
	Doctoral	53		30 (56.6)						27 (50.9)						20 (37.7)						
**Years of clinical practice**							0.772 (2)		.68				6.119 (2)		.047^c^					0.635 (2)		.73
	0-10	89		46 (51.7)						37 (41.6)						38 (37.4)						
	11-20	83		44 (53)						43 (51.8)						31 (37.5)						
	≥21	56		33 (58.9)						35 (62.5)						21 (37.5)						
**Specialty**							1.695 (3)		.64				4.785 (3)		.19					0.928 (3)		.82
	Medical oncology	97		48 (49.5)						44 (45.4)						39 (40.2)						
	Surgical oncology	77		45 (58.4)						41 (53.2)						28 (36.4)						
	Radiation therapy	40		23 (57.5)						25 (62.5)						18 (45)						
	Others	14		7 (50)						5 (35.7)						5 (35.7)						
**Hospital type**							0.563 (1)		.90				0.141 (1)		.71					1.69 (1)		.19
	University hospital	148		79 (53.4)						76 (51.4)						63 (42.6)						
	Nonuniversity hospital	80		44 (55)						39 (48.8)						27 (33.8)						
**IT experience**							0.158 (1)		.69				0.369 (1)		.54					1.12 (1)		.29
	Yes	35		16 (45.7)						16 (45.7)						11 (31.4)						
	No	193		107 (55.4)						99 (51.3)						79 (40.9)						

^a^AI: artificial intelligence.

^b^Percentages expressed with the value in the “Total oncologists, N” column as the denominator.

^c^*P*<.05.

### Oncologists’ View on “The Impact of AI on the Doctor-Patient Relationship”

As for the impact of AI on doctor-patient relationships, a majority (121/228, 53.1%) believed that AI would have a positive impact on the doctor-patient relationship. However, 9.2% (21/228) of the respondents felt that AI could cause trouble and increase disputes between doctors and patients, whereas 2.2% (5/228) believed it would not have any impact. In all, 35.5% (81/228) of the respondents reported that it was hard to judge, meaning they had mixed feelings about the statement (Table 5).

The study revealed that perceptions of AI’s impact on the doctor-patient relationship varied with sex, education, and clinical experience.

Regarding sex, female physicians tended to find it harder to judge than male physicians (39/93, 42% vs 42/135, 31.1%), whereas male physicians were more positive than female physicians (74/135, 54.8% vs 47/93, 50%). However, the difference in proportions was not statistically significant (*P*=.08). Education degree appeared to influence the responses. Those with a bachelor’s degree showed the highest difficulty in making a judgment (26/49, 53%), and the difference in response according to education level was statistically significant (*P*=.03). When analyzing the years of clinical practice, practitioners with 21 or more years of experience had the highest difficulty in making a judgment (28/56, 50%). However, this difference was not statistically significant (*P*=.054). Regarding specialties, there were no significant differences among the responses (*P*=.15). The hospital type did not show any significant differences either (*P*=.42). Lastly, IT experience played a significant role in judgment, with those having IT experience showing more positive responses (25/35, 71%) compared to those without IT experience (96/193, 49.7%). This difference was statistically significant (*P*=.02; Table 6).

**Table 5:** Oncologists’ view on the statement “the impact of AI on the doctor-patient relationship” (N=228).

Response	Oncologists, n (%)
Positive	121 (53.1)
Negative	21 (9.2)
No impact	5 (2.2)
Hard to judge	81 (35.5)

**Table 6:** Oncologists’ characteristics in relation to the statement “the impact of AI^a^ on the doctor-patient relationship.”

Characteristics			Total oncologists, N		Hard to judge, n (%)^b^		Positive, n (%)		Negative, n (%)		No impact, n (%)		Chi-square (*df*)			*P* value	
**Sex**														6.88 (3)			.08
	Male	135		42 (31.1)		74 (54.8)		17 (12.6)		2 (1.5)							
	Female	93		39 (41.9)		47 (50.5)		4 (4.3)		3 (3.2)							
**Education degree**														13.829 (6)			.03^c^
	Bachelor’s	49		26 (53.1)		18 (36.7)		5 (10.2)		0 (0)							
	Master’s	126		41 (32.5)		70 (55.6)		10 (7.9)		5 (4)							
	Doctoral	53		14 (26.4)		33 (62.3)		6 (11.3)		0 (0)							
**Years of clinical practice**														12.378 (9)			.054
	0-10	89		28 (31.5)		55 (61.8)		4 (4.5)		2 (2.4)							
	11-20	83		25 (30.1)		44 (53)		12 (14.5)		2 (2.2)							
	≥21	56		28 (50)		22 (39.3)		5 (8.9)		1 (1.8)							
**Specialty**														13.323 (6)			.15
	Medical oncology	97		33 (34)		56 (57.7)		5 (5.2)		3 (3.1)							
	Surgical oncology	77		26 (33.8)		38 (49.4)		13 (16.9)		0 (0)							
	Radiation therapy	40		18 (45)		19 (47.5)		2 (5)		1 (2.5)							
	Others	14		4 (28.6)		8 (57.1)		1 (7.1)		1 (7.1)							
**Hospital type**														2.796 (3)			.42
	University hospital	148		50 (33.8)		78 (52.7)		17 (11.5)		3 (2)							
	Nonuniversity hospital	80		31 (38.8)		43 (53.8)		4 (5)		2 (2.5)							
**IT experience**														10.233 (3)			.02^c^
	Yes	35		5 (14.3)		25 (71.4)		3 (8.6)		2 (5.7)							
	No	193		76 (39.4)		96 (49.7)		18 (9.3)		3 (1.6)							

^a^AI: artificial intelligence.

^b^Percentages expressed with the value in the “Total oncologists, N” column as the denominator.

^c^*P*<.05.

### Oncologists’ View on “AI Will Replace Doctors”

In terms of acceptance of the statement “AI will replace doctors,” the result indicated mixed opinions. Overall, 23.2% (53/228) strongly disagreed with the statement, 14% (32/228) disagreed, 29.8% (68/228) were neutral, 16.2% (37/228) agreed, and 16.7% (38/228) strongly agreed (Table 7).

The study revealed a diversity of views on AI’s potential to replace doctors, but there were no significant correlations with demographic and professional factors (all *P*>.05).

**Table 7:** Oncologists’ view on the statement “AI^a^ will replace doctors” (N=228).

Response	Oncologists, n (%)
Strongly disagree	53 (23.2)
Disagree	32 (14)
Neutral	68 (29.8)
Agree	37 (16.2)
Strongly agree	38 (16.7)

^a^AI: artificial intelligence.

## Discussion

### Principal Findings

Our survey delved into the many concerns held by oncologists regarding the integration of AI in their respective disciplines. The data procured elucidate an array of apprehensions that can vary significantly in both content and priority, as dictated by multiple factors. These factors are integral to the comprehension and smooth transition of AI adoption within health care. Primary among these apprehensions are misleading diagnoses and treatments by AI, an overreliance on AI potentially diminishing doctors’ capabilities, bias in algorithms and data, issues pertaining to data security and patient privacy, and legal challenges. These issues emerged as the top 5 concerns in this study.

A total of 71.5% (163/228) of respondents expressed anxiety over AI misleading diagnoses and treatments, potentially leading to medical errors and compromising patient safety—a common concern among many physicians [18-20]. Several reasons can underpin this concern. First, AI systems are reliant on training data; biased data lead to a biased AI, potentially resulting in improper diagnosis or treatment recommendations [21]. Second, an AI’s predictions are restricted to its training data. Consequently, incomplete data could lead to inaccurate predictions [22]. Third, the complex and opaque nature of AI systems can hinder users’ comprehension of their conclusions or suggestions, leading to decisions based on incomplete or inaccurate information, thereby potentially harming patients [20]. Fourth, misuse by health care providers lacking appropriate training on AI could lead to suboptimal patient outcomes [23].

Another concern, held by 71% (162/228) of respondents, revolved around an overreliance on AI, leading to a decrease in their diagnostic and treatment capabilities. This aligns with literature emphasizing the necessity of a balanced approach toward incorporating AI into health care, where AI serves as an auxiliary tool, not a replacement for health care professionals [24]. Overreliance can potentially erode critical skills acquired through education, training, and experience [25], as well as lead to complacency and blind trust in AI’s decision-making, consequently compromising patient safety [26].

A majority (123/228, 54%) of respondents expressed concern over data security and patient privacy, resonating with recent studies highlighting similar apprehensions in the era of AI [27,28]. AI systems’ tendency to collect and process vast amounts of patient data makes them an attractive target for hackers, with potential fallout including identity theft, financial loss, reputational damage, and loss of trust [29,30]. This underscores the necessity of robust data protection measures.

Another issue, raised by 54% (123/228) of the participants, pertained to bias in AI’s data and algorithms. These biases can significantly impact health AI, potentially leading to inaccurate diagnoses, missed treatments, and negative outcomes for patients. These biases might also exacerbate existing inequalities [31,32].

Legal ambiguities surrounding AI use were a concern for half (115/228, 50.4%) of the respondents, with an unclear delineation of medical responsibilities posing potential risks. The laws, regulations, and policies governing AI in health care are still evolving, creating uncertainty for health care organizations and providers [33,34]. The European Commission has put forward new regulatory measures for the deployment of “high-risk artificial intelligence,” indicating that the current framework of European fundamental rights already lays down explicit directives for using medical AI, under the title “Fundamental Rights as Legal Guidelines for Medical AI.” Within this context, “obligations to protect” gain significant relevance in the medical field, mandating health care service providers to adopt quality-assurance practices [35]. However, the swift and expansive progression of AI technology and innovations significantly amplifies the threats associated with the underuse of AI. Overregulation threatens to forgo the potential benefits of AI [36].

As for the “black box” phenomenon of AI products, it was a concern for 39.5% (90/228) of participants, mirroring the general call for more explainable and interpretable AI models in the literature [37]. Trustworthy AI must enable professionals to confidently assume responsibility for their decisions, thereby emphasizing the importance of explainable AI techniques. The analytical and clinical effectiveness of AI algorithms requires consistent monitoring. For effective oversight, both explainability and causality must be evident. Experts require proof of explainability and causality to responsibly manage their roles. Therefore, AI must integrate causality assessments to uphold the standard of its explainability [38].

The fact that AI lacks empathy, as noted by 36.8% (84/228) of respondents, is a recurring theme in AI ethics discussions, underlining the irreplaceability of human touch in medical care [39]. The complex operation of AI products (38/228, 16.7%), business model issue (58/228, 25.4%), and poor integration with existing workflows highlight a need for more user-friendly AI solutions that integrate well with health care systems [40]. Interestingly, a small proportion (7/228, 3.1%) of respondents did not perceive any risks associated with AI. The variability in perceptions could be due to differences in understanding, knowledge, and exposure to AI among the respondents [41].

Concerns regarding AI are shaped by a complex blend of demographic, professional, and regional variables. The apprehensions of physicians in their later career stages might be influenced by their technological fluency and privacy concerns [1]. Early-career doctors, who might be more familiar with digital technology, may be more accepting of AI than late-career doctors, who might prefer traditional methods, and showed less concern about AI. Sex differences were noticeable, with female physicians often expressing ethical concerns, whereas male physicians focused on the potential applications and benefits of AI [42]. Higher education can provide knowledge, critical thinking skills, technology exposure, and learning confidence that make individuals more receptive to emerging technologies such as AI, leading to greater trust and acceptance and thus less concern about AI [43,44]. This allows individuals to evaluate the potential risks and benefits of AI more effectively, rather than simply fearing the unknown. However, there are likely other mediating factors, and more research is needed. In our study, we did not find a significant difference in medical specialty, geographic practice location, professional experience, and cultural background that also significantly influenced doctors’ concerns [45]. Acknowledging these intricacies is essential for effective and empathetic AI integration.

Our research also suggests that IT experience makes a difference. Oncologists with IT experience might have a better understanding of the capabilities and limitations of AI, making them more confident in integrating it into their practice. On the other hand, those without IT experience may have apprehensions due to unfamiliarity with the technology.

AI introduces novel challenges for the doctor-patient relationship, as it carries the potential to revolutionize modes of clinical interaction. Consequently, the doctor-patient relationship may evolve from a dyad to a triad, encompassing the doctor, patient, and AI [46]. AI’s role in medicine can instigate a positive shift in the patient-physician relationship. It has been indicated that AI can positively impact doctor-patient relationships, particularly by serving in an assistive role and enhancing medical education [47]. However, its impact on clinical practice and the doctor-patient relationship remains largely undetermined. The effect is likely to vary based on AI’s specific application and use context. AI might also result in a lower standard of care, characterized by fewer personal, face-to-face interactions [48].

Most oncologists surveyed recognize AI’s potential to positively influence the doctor-patient relationship, especially in terms of enhancing patient understanding. Oncologists with higher educational degrees and IT experience tended to have a more positive view of AI. There was also a slight sex difference, with male oncologists appearing slightly more positive toward AI’s impact. However, apprehensions still existed, and these appeared to be influenced by factors such as sex, educational background, and years of clinical practice. This highlights the necessity for nuanced, demographic-specific strategies when incorporating AI into health care practices, to address diverse concerns and expectations.

The idea of AI replacing doctors has been the subject of numerous discussions, studies, and debates in recent years. Based on the study data, the conclusion is that oncologists showed mixed responses toward the statement “AI will replace doctors.” Overall, most oncologists, regardless of sex, age, education degree, years of clinical practice, specialty, type of hospital, or IT experience, tended to be neutral on the question of AI replacing doctors. There were no statistically significant differences in views based on the analyzed factors, suggesting that other factors not captured in this study might be influential, or that views on AI’s capacity to replace doctors were generally ambivalent or uncertain within this professional group. Some scholars and practitioners argue that AI has the potential to outperform humans in some areas of medicine, particularly in tasks involving data analysis and interpretation, such as radiology, pathology, and genomics [49-51]. Researchers argue that AI currently lacks the generalized intelligence, emotional skills, reasoning capacity, and societal trust needed to fully replace human physicians [52-56].

Despite the differing views, it is apparent that the medical community is not widely endorsing the notion of AI replacing doctors as of now. It is generally agreed upon in the medical community that AI should be used as a tool to assist health care professionals and work in collaboration, rather than replace them [24]. It reflects a pragmatic approach, recognizing the potential of AI in enhancing health care delivery while valuing the irreplaceable aspects of human medical practice.

### Suggestion

To effectively address the concerns raised by oncologists about the use of AI in health care, it is essential for AI stakeholders, designers, and researchers to focus on a comprehensive strategy encompassing critical actions.

Educating health care professionals about AI’s capabilities and limitations is vital to prevent overreliance and foster a balanced approach where AI acts as a supportive tool rather than a replacement. Education was identified as a priority to prepare clinicians for the implementation of AI in health care [45].

Encouraging multidisciplinary collaboration among AI researchers, health care professionals, ethicists, and policy makers can address the complex challenges of AI integration, ensuring responsible and ethical development and deployment [57].

The AI vender must prioritize transparency and explainability of AI systems to demystify their operations for clinicians, thereby tackling the “black box” issue [58].

Emphasizing human-centered design and empathy is crucial; AI tools should be developed with health care professionals’ involvement to ensure that they address clinical needs and seamlessly fit into existing workflows, thus enhancing user experience and bridging the emotional gap between AI and humans. Addressing bias through rigorous testing and validation across diverse data sets is essential to prevent perpetuating existing inequalities and ensure that AI applications are equitable [59].

By concentrating on these targeted actions, AI stakeholders can substantially contribute to the responsible and effective integration of AI in oncology, ultimately enhancing patient outcomes and fostering trust in AI-assisted health care.

### Limitations

This study has several limitations. First, this study is constrained by its small sample size, which diminishes its statistical power and heightens the potential for error. A future study will aim to expand participant recruitment and increase the sample size to mitigate this issue. Second, individuals presented with a survey concerning a topic they find engaging are more likely to participate compared to those who perceive the topic as less interesting [60]. Third, we referred to a validated questionnaire that was adapted from many studies, which was developed by doctors rather than AI experts. Some items were not considered due to the experts’ suggestion. Thus, our study only provides information on physicians’ concern of AI.

### Conclusion

In conclusion, this study has highlighted the primary concerns of oncologists regarding AI, underscoring significant implications for stakeholders in the health care sector. To successfully integrate AI into health care, it is imperative to address these concerns through a unified effort involving policy makers, AI developers, health care professionals, and legal experts. A comprehensive strategy, encompassing transparent and understandable AI systems and human-centered design, addressing biases, and educating health care providers on AI’s capabilities and limitations, is essential. Such a collaborative and multidisciplinary approach will pave the way for AI to become a valuable ally in health care, thus enhancing patient care and outcomes.

## Appendix

Multimedia Appendix 1 The questionnaire of this study.

## Abbreviations

AI: artificial intelligence

## Data Availability

The data sets generated during and/or analyzed during this study are available from the corresponding author on reasonable request.
