# Supplementary material for: Chinese Oncologists’ Perspectives on Integrating AI into Clinical Practice: Cross-Sectional Survey Study
Source: JMIR Form Res. 2024 Jun 5;8:e53918. doi: 10.2196/53918 (PMC11187515; doi:10.2196/53918)
Supplement: Multimedia Appendix 1 [file formative_v8i1e53918_app1.docx]

Health AI research Questionnaire （The original is in Chinese）

**Characters**

1. Gender 【Single-choice】

○ Male

○ Female

1. Education 【Single-choice】

○ Undergraduate

○ Master's degree

○ Doctorate

1. Your clinical practice time 【Single choice】

○ ~ 5 years

○ 6-10 years

○ 11-15 years

○ 16-20 years

○20 ~years

1. Your age

○ ~30

○ 31-40

○ 41-50

○ 51-60

1. What is your department? [Single-choice】

○ Medical Oncology

○ Surgical oncology

○ Radiation therapy

1. Your hospital type 【Single choice

○ University hospital

○ Non-University hospital

1. Have you been involved in the design or development of IT products (HIS systems, electronic medical records EMR, medical artificial intelligence products, etc.)? [Single choice question

○Yes

○ None

**Knowledge, perceptions and willingness to AI**

1. What do you think "artificial intelligence" is? (Single choice) [Multiple choice

□ Computer, Internet

□ Algorithms, data

□ Robotics

□ I've heard of it, but I'm not sure what AI is exactly, it's very abstract.

□ Have a good understanding of AI and a clear definition of it

1. What are the medical AI products that you are currently exposed to or use in your clinical work? (Multiple choice) [Multiple choice

□ Medical image AI-assisted diagnosis category (X-ray, CT, MRI, pathology, ultrasound, ECG, etc.)

□ Clinical decision support category (medical advice prompts, diagnostic prompts, treatment plan recommendations, radiotherapy plan development, surgical navigation, etc.)

□ Disease risk prediction (genetic testing, VTE assessment, drug efficacy, etc.)

□ Medical service category (voice and text entry, intelligent consultation, etc.)

□ Medical Robotics

□ Research (basic medicine, clinical medicine analysis related products)

□ Not currently in use

□ Other

1. What is your current knowledge of and exposure to ChatGPT? (Single choice)

○ Have not heard of ChatGPT.

○ Knows about ChatGPT, but has no need or desire to use it.

○ Would love to try using ChatGPT, but haven't had a chance or aren't sure how.

○ Have used ChatGPT or ChatGPT embedded products.

1. How much do you know about Artificial Intelligence and your willingness to learn? (1 star indicates that you don't know, don't want to or shouldn't, 5 stars indicates that you know it very well, are willing to or should) 【Scoring Question】 (Please fill in 1-5 numbers to score)

Knowledge of the terms Artificial Intelligence, Machine Learning, Deep Learning:: 1 is not agree,5 is strongly agree Your rating is ____

Knowledge of the limitations of artificial intelligence:: 1 is not agree,5 is strongly agree Your rating is ____

Degree of personal subjective willingness to learn about AI-related knowledge and applications:: 1 is Strongly disagree,5 is strongly agree Your rating is ____

Should clinicians be trained in knowledge and applications related to artificial intelligence:: 1 is not agree,5 is strongly agree Your rating is ____

1. How much do you perspective of Medical Artificial Intelligence. (1 star indicates disapproval, 5 stars indicates strong approval) 【Scoring Question】 (Please fill in 1-5 numbers to score)

Artificial Intelligence is beneficial to the healthcare industry :: 1 out of 5 is Strongly disagree,5 out of 5 is strongly agree Your rating is ____

Your trust in current medical AI technology:: 1 is Strongly disagree,5 is strongly agree Your rating is ____

Your acceptance of current healthcare AI technology:: 1 is Strongly disagree,5 is strongly agree Your rating is ____

Current AI has exceeded the average clinician's ability to treat patients:: 1 is Strongly disagree,5 is strongly agree Your rating is ____

1. Your outlook on artificial intelligence technology. (1 star indicates disapproval, 5 stars indicates strong approval) 【Scoring Question】 (Please fill in 1-5 numbers to score)

Artificial Intelligence is the future of the healthcare industry :: 1 for Strongly disagree,5 for strongly agree Your rating is ____

Artificial Intelligence will revolutionize the healthcare system :: 1 is not agree,5 is strongly agree Your rating is ____

1. Your degree of willingness to use AI-related products. (1 star means not willing, 5 stars means very willing) 【Scoring Question】 (Please fill in 1-5 numbers to score)

I can use Artificial Intelligence related products:: 1 out of 5 are Strongly disagree,5 out of 5 are strongly agree Your rating is ____

would like to use as many AI-related products as possible 1 is Strongly disagree,5 is strongly agree Your rating is ____

**Promotion factors**

1. What are the positive factors affecting your use of AI-related products in oncology clinical work? (Multiple choice) [Multiple choice question

□ Improve the standardization and precision of medical treatment.

□ Reduce misdiagnosis and underdiagnosis.

□ Improve the efficiency of clinical and scientific work.

□ Helps doctors and patients to communicate and increases patients' trust in doctors.

□ Personal interest and willingness to try new technologies.

□ Used at the request of leaders and under the influence of colleagues.

□ Artificial intelligence products are easy to use.

□ Promotion of use under the influence of an epidemic.

□ Other

**Concern**

1. What are your concerns about AI medical products? (Multiple choice) [Multiple choice question

□AI misleads diagnosis and treatment

□ Over-reliance on AI

□ data and algorithmic bias.

□ data security and patient privacy.

□ Artificial intelligence lacks human emotion.

□ T "black box" phenomenon f

□ AI business model issue could not promotion

□ Not easy to use and not well integrated with clinical workflow

□ Laws, regulation, and policies lagging behind

□ No any Risks

□ Other

1. The impact of artificial intelligence on the doctor-patient relationship: (Single choice)

○ Positive

○ No significant impact.

○ Negative

○ Hard to judgement

1. Artificial Intelligence Will Replace Doctors : 1 is strongly disagree,5 is strongly agree Your rating is ____
2. What do you think artificial intelligence technology will bring to oncology clinics in the future? 【Fill in the blank】 （Optional）

________________________
